# IL-13 Augments Histone Demethylase JMJD2B/KDM4B Expression Levels, Activity, and Nuclear Translocation in Airway Fibroblasts in Asthma

**DOI:** 10.1155/2021/6629844

**Published:** 2021-02-22

**Authors:** Khuloud Bajbouj, Mahmood Y. Hachim, Rakhee K. Ramakrishnan, Huwaida Fazel, Jumana Mustafa, Shahed Alzaghari, Mahmoud Eladl, Jasmin Shafarin, Ronald Olivenstein, Qutayba Hamid

**Affiliations:** ^1^College of Medicine, University of Sharjah, Sharjah, UAE; ^2^Sharjah Institute for Medical Research, University of Sharjah, Sharjah, UAE; ^3^College of Medicine, Mohammed Bin Rashid University of Medicine and Health Sciences, Dubai, UAE; ^4^Meakins-Christie Laboratories, McGill University, Montreal, QC, Canada

## Abstract

**Purpose:**

Asthma is one of the most common obstructive pulmonary diseases worldwide. Epigenetic alterations, including DNA methylation and histone modifications, have been reported to contribute to asthma pathogenesis. Since the inflammation mediator and remodeling trigger, IL-13, is known to play a central role in the pathophysiology of asthma, this study was aimed to identify novel IL-13-regulated epigenetic modifiers in asthma that may contribute to subepithelial fibrosis.

**Methods:**

Publicly available transcriptomic datasets from Gene Expression Omnibus (GEO) were used to identify differentially expressed genes on an epigenetic level upon IL-13 exposure in lung fibroblasts. Bronchial fibroblasts isolated from healthy and asthmatic individuals were assessed for the gene and protein expression levels of the identified gene at baseline and upon IL-13 treatment using qRT-PCR and western blotting, respectively. Its subcellular localization and tissue distribution were examined in bronchial fibroblasts as well as bronchial biopsies by immunofluorescence and immunohistochemical analysis, respectively.

**Results:**

Bioinformatic analysis revealed the differential expression of the histone demethylase JMJD2B/KDM4B, a well-known epigenetic modulator that leads to the demethylation of different lysine residues on histones, in IL-13-treated lung fibroblasts. The baseline expression levels of JMJD2B were higher in asthmatic fibroblasts and in bronchial biopsies in comparison to healthy ones. There was also an increase in JMJD2B activity as evidenced by the demethylation of its downstream target, H3K36me3. Furthermore, IL-13 stimulation induced JMJD2B expression and further demethylation of H3K36me3 in asthmatic fibroblasts. This was accompanied by increased translocation of JMJD2B into the nucleus.

**Conclusion:**

This study highlights the novel pathological involvement of the histone demethylase JMJD2B/KDM4B in asthmatic airway fibroblasts that are regulated by IL-13. *Clinical implications*. Given that there is no single therapeutic medicine to effectively treat the various subtypes of asthma, this study provides promising insights into JMJD2B as a new therapeutic target that could potentially improve the treatment and management of asthma.

## 1. Introduction

Asthma is an inflammatory disease of the airways, characterized by different degrees of obstruction that results from airway remodeling. An inflammatory immune response is triggered by the exposure of asthmatic airways to allergens, resulting in the activation of T helper type 2 (Th2) lymphocytes. These Th2 cells release cytokines such as interleukin- (IL-) 4, IL-5, IL-9, and IL-13 that in turn regulate both airway inflammation as well as airway remodeling. IL-13 in particular plays a pivotal role in the regulation of the allergic diathesis.

Subepithelial fibrosis is a feature of airway remodeling that is associated with an aberrant fibroblast phenotype. There are reported differences in the morphology and cytoskeletal architecture between asthmatic and nonasthmatic fibroblasts. In contrast with nonasthmatic normal fibroblasts, the asthmatic fibroblasts form thick and aligned ventral stress fibers that are accompanied by focal adhesions [1]. Asthmatic fibroblasts also exhibit a synthetic phenotype with increased expression of extracellular matrix components, such as type 1 collagen, proteoglycans, versican, hyaluronan, fibronectin, decorin, and tenascin C, and their increased secretion into the surrounding environment. Many studies have shown the increased potential of asthmatic fibroblasts to differentiate into myofibroblasts in comparison to nonasthmatic fibroblasts [2, 3]. IL-13 is an important contributor to subepithelial fibrosis by exerting its action on fibroblasts. It modulates the remodeling of the subepithelial basement membrane by stimulating increased fibroblast proliferation, myofibroblastic differentiation, and extensive collagen deposition leading to thickening and narrowing of the airways [2, 3].

Epigenetic processes, including DNA methylation, histone acetylation, microRNA expression, and chromatin alterations, alter gene transcription without a change in the DNA nucleotide sequence. Epigenetic modulations have been implicated to play a role in the pathogenesis of respiratory diseases. When the normal epigenetic regulation is disturbed by environmental exposures, Th1/Th2 balance can be affected and can thereby contribute to the pathogenesis of asthma and COPD [4]. Some of these environmental triggers linked with the epigenetics of asthma, and specifically with DNA methylation, include low birth weight [5], gestational folate levels [6], socioeconomic position [7], and smoking [8], to name a few. IL-13 is known to induce epigenetic changes in asthmatic airways and to alter transcriptional gene regulation [9]. Even a single exposure of IL-13 may induce DNA methylation changes in airway epithelial cells and contribute to fibrosis and asthmatic phenotype [10]. Further, the epigenetic regulation of IL-13-mediated collagen deposition by fibroblasts suggests an epigenetic fibrotic response of fibroblasts to IL-13 exposure [11]. Since the involvement of IL-13-mediated epigenetic regulation of subepithelial fibrosis in asthma is not clearly understood, we employed bioinformatics to identify novel IL-13 regulated epigenetic modifiers in asthma.

Jumonji C (JmjC) is a group of histone demethylases that regulates gene transcription epigenetically. Different JmjC domain-containing proteins have been shown to play a role in cancer regulation and progression and have been used as biomarkers in some types of cancer [12]. JMJD2B, formerly known as KDM4B, is a member of the JmjC protein family that activates gene expression through demethylation of di- and trimethylated histones, such as H3K9me2/me3, H3K36 me2/me3, and H3K4 me2/me3 histones. It has been shown to play a role in the progression of both lung cancer and bladder cancer cell proliferation [12, 13]. The activation of the histone demethylase JMJD2B, a gene that is expressed in fibroblasts, leads to the demethylation of different lysine residues on histones that may activate genes responsible for fibrosis [12].

Asthma is a multifactorial disease that involves subepithelial fibrosis and associated narrowing of the airways. Here, we have identified the novel pathological involvement of the histone demethylase JMJD2B/KDM4B in asthmatic airway fibroblasts that is further regulated by IL-13. We show that the disease pathology occurs at the molecular (IL-13 expression), genetic (JMJD2B activation), epigenetic (H3K36me3 demethylation), and cellular (fibroblast activation) levels.

## 2. Materials and Methods

### 2.1. Identifying Core Differentially Expressed Genes in IL-13 Treated versus Untreated Adult Lung Fibroblasts

The publicly available transcriptomic datasets from Gene Expression Omnibus (GEO) were filtered to search for a dataset that includes primary lung fibroblasts treated with IL-13 versus control without IL-13 stimulation. One dataset (GSE43515) included the needed setting, where adult lung fibroblasts were treated with or without IL-13. Raw CEL files were extracted and subjected to Preprocessing Quality Control, normalization, and filtering. Differentially expressed genes (DEGs) were then identified based on Gene Set Enrichment Analysis (GSEA). To filter out nonvariant genes between IL-13-treated and vehicle controls-treated fibroblasts, a combination of noise and variance filtering was applied. Only probes with a value of 100 or higher in the MAS5 dataset in all 12 samples were selected. The probes that passed the first filter, then, are subjected to the coefficient of variation (CV) filter using their gcRMA expression intensities. Probes with a CV value of 10-50% across all samples were considered to be variant and thus selected. CV was calculated as the mean/standard deviation of each gene across all samples.

### 2.2. In Silico Validation of Initially Identified DEGs in a Different Dataset

To confirm that these identified genes are differentially expressed in response to IL-13 in lung fibroblasts, another independent dataset (GSE56338) was used for *in silico* validation. In this setting, human fibroblasts were stimulated with IL-13 and compared to media and pretreatment controls. 2 cell passages were employed with each including 2 independent experiments.

### 2.3. Identifying Regulatory Programs on the Epigenetic Level for the Identified DEGs

We used Enrichr: a comprehensive gene set enrichment analysis web server library which contains extensive processed ChIP seq data from Roadmap Epigenomics Project to associate detected peaks near genes to identify gene regulatory programs on the epigenetic level. The DEGs identified earlier were uploaded to Enrichr online tool (https://maayanlab.cloud/Enrichr/enrich#), and Epigenomics Roadmap HM ChIP-seq results were downloaded. Only sets with adjusted *p*-value < 0.05 were selected that were related to fibroblasts.

### 2.4. Enriched Ontology Clustering for the Identified Genes

Enriched Ontology Clustering for the identified genes was performed using Metascape (http://metascape.org/gp/index.html#/main/step1) [12–15].

### 2.5. Cell Culture and IL-13 Stimulation

Human-derived fibroblasts from healthy and asthma subjects were maintained in DMEM medium supplemented with 2 *μ*g/mL of insulin, 1 mM of sodium pyruvate, 1 mM of nonessential amino acids, 4 mM of glutamine, 10% fetal calf serum, and antibiotics (penicillin/streptomycin) at 37°C and 5% CO_2_. Cells were seeded at 0.5 − 1 × 10^5^ cells/mL in 25 cm^2^ flasks. At ~70% confluency, cells were stimulated with 20 ng/ml of IL-13 (ProSpec). Control cell cultures were either left untreated or treated with equal volumes of DMSO as vehicle.

### 2.6. Western Blotting Analysis

Cells were lysed in ice-cold RIPA buffer (Abcam) containing protease inhibitor cocktail tablets (Sigma). Whole cell lysate protein concentrations were quantified using the standard Bradford method (Bio-Rad). Lysate aliquots containing 30-50 *μ*g of protein were separated by 12% sodium dodecyl sulfate–polyacrylamide gel electrophoresis (SDS-PAGE) and transferred onto a nitrocellulose membrane (Bio-Rad). The membrane was then blocked by 5% skimmed milk powder for 1 h at room temperature, washed with TBST, and reacted with primary immunoglobulin G (IgG) unlabeled primary antibodies; anti-JMJD2B (Abcam), various modified and total histones (Cell signaling), *β*-actin (Sigma), at 1 : 1000 dilution overnight at 4°C. The secondary (anti-mouse and anti-rabbit) antibodies (Cell Signaling) were then reacted with the membrane at 1 : 1000 dilution for 1 h at room temperature. Chemiluminescence was detected using the ECL kit (Thermo Scientific Pierce). Protein band quantification was carried out using the Bio-Rad Image Lab software (ChemiDoc™ Touch Gel and Western Blot Imaging System; Bio-Rad). *β*-Actin was used as a normalization control.

### 2.7. Quantitative Real-Time PCR

The cDNA was synthesized from 1 *μ*g of total RNA using the QuantiTect Reverse Transcription Kit (Qiagen) according to the manufacturer's protocol. qPCR was performed using 1 : l of complementary DNA (cDNA), specific primers for each gene, SYBR® Green I, and an iCycler Thermal Cycler. Expression levels of target human gene JMJD2B (F:5′-GGACTGACGGCAACCTCTAC-3 ′, R:5 ′-CGTCCTCAAACTCCACCTG-3 ′) was normalized to GAPDH expression (F:5 ′-CCAGGTGGTCTCCTCTGACTTC-3 ′, R:5 ′-TCATACCCAGGAAATGAGCTTGACA-3 ′).

### 2.8. Immunofluorescence Staining

Cells were seeded at 10^4^ cells/ml on sterile poly-L-lysine-coated glass cover slips in 6-well culture plates and cultured overnight. Cells were starved for 12 hr prior to IL-13 stimulation as described previously. Cells on cover slips were then washed with PBS and fixed with 4% paraformaldehyde for 15 min at room temperature and treated with 0.1% Triton X-100 for 10 min. Fixed and permeabilized cells were blocked with BSA at 3% for 1 hr, rinsed with 1X PBS and incubated with unlabeled JMJD2B primary antibody (Abcam) overnight at 4°C. Cells were then washed with 1X PBS and reacted with the Alexafluor® 488- or Alexafluor® 680-labeled secondary antibody (Abcam) for 1 hr at 37°C. Genomic DNA was stained with 4′,6′-diamidino-2-phenylindole (DAPI) (Invitrogen) according to manufacturer's instructions. Slides were visualized by fluorescence microscopy using an Olympus BX51 fluorescence microscope (Olympus Corporation, Tokyo, Japan).

### 2.9. Immunohistochemical Staining

Immunohistochemistry was performed as previously described [14]. Briefly, the bronchial biopsy sections were deparaffinized using xylene, and antigen retrieval was performed in Tris-EDTA buffer with pH 9. JMJD2B primary antibody (Cell signaling Technology) was used at a dilution of 1 : 50, followed by the corresponding biotinylated secondary antibody. Color development was achieved using HRP/DAB Detection IHC kit (abcam). Counterstaining was done using hematoxylin, and the slides were rinsed and mounted with DPX mounting medium.

### 2.10. Statistical Analysis

All graphical data was analyzed using GraphPad Prism 5 (GraphPad Software Inc., La Jolla, CA, USA), and unpaired *t*-test was used to generate *p*-values for comparisons between groups in each data set.

## 3. Results

### 3.1. Bioinformatic Analysis Revealed Differential Expression Pattern of JMJD2B upon IL-13 Stimulation

1362 genes were identified to be variable between the IL-13-treated and untreated groups of fibroblasts indicating their role in the response of lung fibroblasts to IL-13. These genes were further processed to identify their shared pathways and common regulators.

#### 3.1.1. The Identified Genes Are Regulated or Affected by Histone (H2 and H3) Modification

The identified genes showed overlap with gene sets that are regulated or affected by histone modifications as studied in IMR90 (normal lung fibroblast cell line from a human female). Histone (H2 and H3) modifications (H2BK12ac, H2BK20ac, H2BK120ac, H2BK15ac, H3K9ac, H4K8ac, H3K18ac, and H3K27ac) were detected as shown in table (Supplementary (available here)).

#### 3.1.2. Four DEGs (JMJD2B, JMJD2C, PTDSR, and JMJD3) Were Involved in Histone Trimethylation and or Demethylation

As our results showed that the DEGs between IL-13-treated and IL-13-untreated fibroblasts are related to histone modifications, we then searched which among these genes are related to histone modifications in their Biological Process (GO) using a metascape database. 4 genes were found to be involved in histone trimethylation and or demethylation, namely JMJD2B, JMJD2C, PTDSR, and JMJD3 (Table 1).

#### 3.1.3. IL-13 Upregulated JMJD2B Expression in Adult Lung Fibroblasts

**Figure 1:**
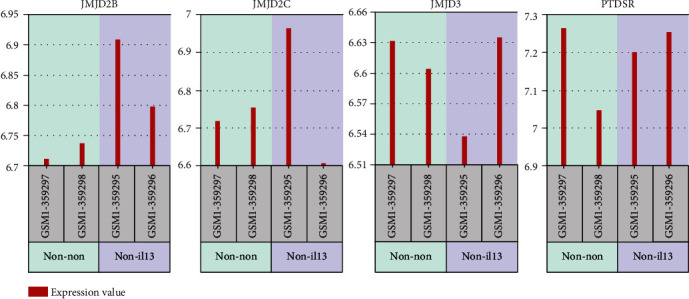
Normalized gene expression of four DEGs genes (JMJD2C, JMJD2B, PTDSR, and JMJD3) was involved in histone trimethylation and or demethylation as extracted from GSE56338 dataset. Adult lung fibroblasts (passage 6) treated with IL-13 (non-il13) compared to the same cells treated with media only (non-non).

To understand the effect of IL-13 on the expression of JMJD2B, JMJD2C, JMJD3, and PTDSR in lung fibroblasts, another dataset (GSE56338) was used for *in silico* validation of earlier results. We extracted the normalized gene expression value for the 4 genes and compared the fibroblasts treated with IL-13 with those left untreated. Compared to media-treated cells, IL-13-treated fibroblasts showed upregulation of JMJD2B expression (Figure 1).

### 3.2. JMJD2B Expression and Activity Is Higher in Asthmatic-Derived Airway Fibroblasts

**Figure 2:**
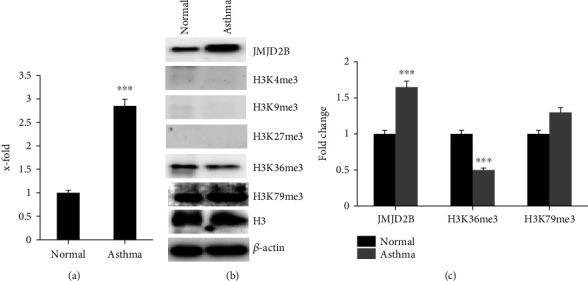
Baseline differential levels of histone demethylase JMJD2B and downstream targets in healthy and asthmatic fibroblasts. (a) qRT-PCR analysis of JMJD2B expression levels. (b) Western bolt analysis showing JMJD2B and trimethylated histones H3 lysine residues at K4, K9, K27, K36, and K79 protein levels in the fibroblasts. (b) Calculated mean ± SD fold change in protein-expression levels in normal and asthma fibroblasts based on two separate experiments. ^∗∗∗^*p* < 0.01, determined using unpaired two-tailed Student *t*-test. Representative immunoblots depicting protein levels normal and asthma fibroblasts where *β*-actin was used as loading control.

In order to compare the gene expression levels of JMJD2B in the fibroblasts obtained from the airway submucosa of healthy and asthmatic individuals, quantitative real-time PCR was carried out. The gene expression of JMJD2B was higher in asthmatic-derived airway fibroblasts than in healthy airway fibroblasts (Figure 2(a)). This further validated in part the results of the bioinformatic analysis indicating a pathological role of JMJD2B in asthmatic fibroblasts.

Consequently, a western blot was run and developed, comparing JMJD2B and the trimethylated histone H3 at lysine residues K4, K9, K27, K36, and K79, together with housekeeping gene *β*-actin, in both healthy and asthmatic fibroblasts. In agreement with the gene expression levels, increased protein expression of JMJD2B was observed in the asthmatic fibroblasts (Figure 2(b)). Considering the demethylation potential of JMJD2B, western blotting revealed that H3K36me3 levels were significantly reduced in correlation with higher JMJD2B in asthmatic fibroblasts (Figure 2(c)). No significant H3 demethylation was detected at positions K4, K9, K27, and K79 in asthmatic fibroblasts when compared to healthy. These results indicate the presence of increased JMJD2B expression and activity levels in the asthmatic airway fibroblasts than in the healthy airway fibroblasts.

### 3.3. IL-13 Augmented JMJD2B Expression and Activity in Asthmatic Fibroblasts

**Figure 3:**
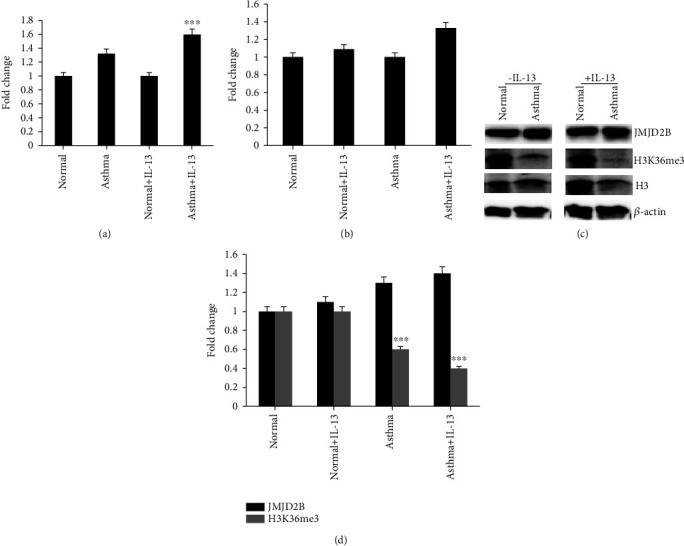
JMJD2B and its downstream target levels after IL-13 stimulation in normal and asthmatic fibroblasts. (a) qRT-PCR analysis of JMJD2B expression levels upon IL-13 stimulation in normal and asthma fibroblasts. (b) qRT-PCR analysis of JMJD2B expression levels comparing fibroblasts with or without IL-13 stimulation in normal and asthma fibroblasts. (c) Western bolt analysis showing JMJD2B and trimethylated histones H3 lysine residue at K36 protein levels in the fibroblasts upon IL-13 stimulation. (d) Calculated mean ± SD fold change in protein-expression levels comparing fibroblasts with or without IL-13 stimulation in normal and asthma fibroblasts based on two separate experiments. Graphical data are represented as mean ± SEM. ^∗∗∗^*p* < 0.01, determined using unpaired two-tailed Student *t*-test. Representative immunoblots depicting protein levels normal and asthma fibroblasts where *β*-actin was used as loading control.

Bioinformatic analysis suggested IL-13 mediated epigenetic regulation of lung fibroblasts through JMJD2B expression. Furthermore, IL-13 is known to induce fibrosis by increasing the activity of fibroblasts, and we investigated if this involved promoting the expression of the JMJD2B gene in asthmatic fibroblasts. Therefore, the fibroblasts were stimulated with IL-13, and the subsequent expression of JMJD2B was measured through quantitative real-time PCR and western blotting, and the activity of JMJD2B was measured through western blotting. In comparison to the healthy fibroblasts, the increased gene expression of JMJD2B in asthmatic fibroblasts was further boosted in the presence of IL-13 (Figure 3(a)). After IL-13 stimulation, the increase in JMJD2B gene expression was more pronounced in asthmatic fibroblasts as compared to healthy ones (Figures 3(a) and 3(b)). A slight increase was also evident in JMJD2B protein levels of asthma cells incubated with IL-13 when compared to the control counterparts (Figure 3(c)). Moreover, since this gene codes for a demethylase enzyme, the levels of methylated histones were measured before and after IL-13 stimulation in both cell lines. A significant decline in H3 methylation was noted at position K36 in asthmatic fibroblasts upon incubation with IL-13 (Figure 3(d)). These results suggest the possibility of IL-13 to enhance JMJD2B expression and subsequent demethylation activity to a significant extent in asthmatic fibroblasts than in healthy fibroblasts.

### 3.4. IL-13 Enhanced JMJD2B Nuclear Translocation

**Figure 4:**
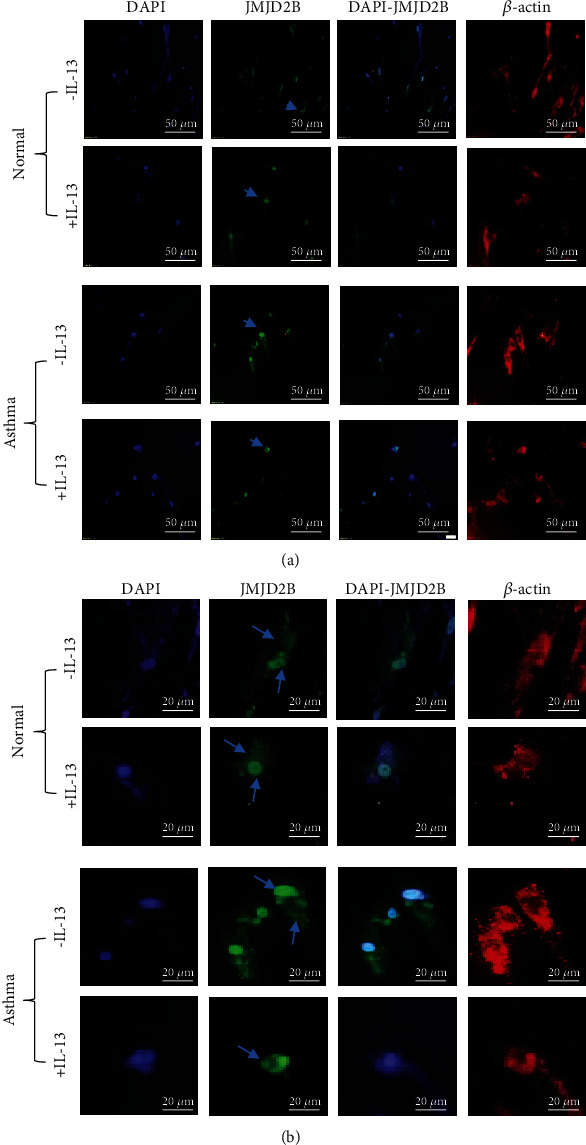
JMJD2B subcellular localization after IL-13 stimulation in normal and asthmatic fibroblasts. Immunofluorescence staining of in normal and asthmatic fibroblasts stimulated with IL-13 that were stained for DNA (DAPI; blue), JMJD2B (green), *β*-actin (red). The images were observed under a microscope at 20x magnification (a) and at 40x magnification (b). Arrows indicate subcellular localization of JMJD2B in fibroblasts.

Considering its histone demethylation activity, JMJD2B mostly has a nuclear localization. It tends to concentrate more in the nucleus when its activity increases. Immunofluorescence staining of both healthy and asthmatic fibroblasts was performed before and after incubation with IL-13 to detect the subcellular localization of JMJD2B. In addition to increased JMJD2B immunoreactivity in the asthmatic fibroblasts, JMJD2B concentrated more in the nucleus following IL-13 stimulation, reflecting increased activity in asthmatic fibroblasts (Figures 4(a) and 4(b)). The change in subcellular localization was less evident in healthy fibroblasts.

### 3.5. Enhanced Nuclear JMJD2B Expression in Asthmatic Bronchial Biopsies

**Figure 5:**
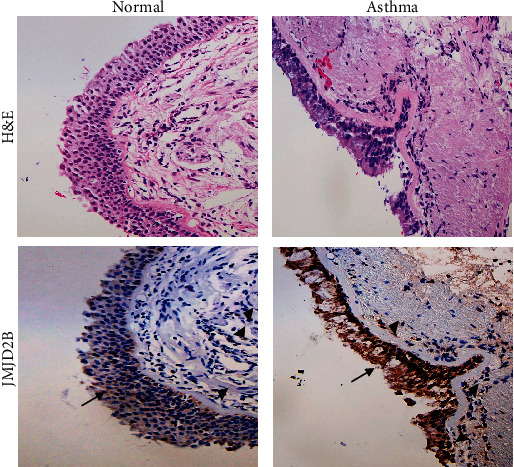
JMJD2B expression in asthmatic bronchial biopsy tissues. Representative bronchial biopsy sections from healthy control showing weak and asthmatic showing moderate to strong JMJD2B protein expression. Representative images for H&E staining taken at 10x magnification and IHC for JMJD2B taken at 20x magnification showing developed with 3,3′- diaminobenzidine (brown). Nuclei were counterstained with hematoxylin (blue). Arrows refer to bronchial epithelium. Arrowheads refer to fibroblasts.

In order to validate our results thus far, we performed immunohistochemical staining of endobronchial biopsy tissue from asthmatic and healthy individuals, which further confirmed the nuclear localization of JMJD2B in asthmatic samples. In accordance with the above results, intense immunostaining of JMJD2B was observed in the epithelial and submucosal compartments of asthmatic specimens when compared to control. Furthermore, nuclear staining of JMJD2B can be observed in both the ciliated epithelium as well as in fibroblasts in the asthmatic tissue specimen when compared to the control tissue (Figure 5).

## 4. Discussion

Our study identified the dysregulation of a novel gene, JMJD2B, in asthmatic fibroblasts. Bioinformatic analysis suggested the differential expression of JMJD2B/KDM4B, a histone demethylase, in lung fibroblasts upon IL-13 stimulation. This was validated in asthmatic fibroblasts and bronchial biopsies, where JMJD2B expression levels were elevated in asthmatic specimens at baseline in contrast to their healthy counterparts. This was found to be further enhanced upon IL-13 stimulation. IL-13 stimulation also resulted in increased JMJD2B translocation into the nucleus and further demethylation of H3K36me3, reflecting its possibly increased activity in asthmatic fibroblasts.

IL-13 is a well-known mediator of subepithelial fibrosis and inducer of epigenetic changes in asthmatic airways. Since IL-13 plays a central role in the pathogenesis of asthma, we used bioinformatics to identify novel IL-13- regulated epigenetic modifiers in asthma. Our analysis identified the involvement of a novel gene, namely JMJD2B/KDM4B, in the pathogenesis of subepithelial fibrosis in asthma that is responsive to IL-13 stimulation.

In order to validate our bioinformatic findings, we used primary bronchial fibroblasts from asthmatic and healthy individuals. Our results demonstrated the increased expression and activity of JMJD2B at baseline in asthmatic fibroblasts compared to its healthy counterparts, which was evidenced by the demethylation of its downstream target H3K36me3 (Figure 2(b)). While upregulated JMJD2B expression has been implicated in the pathogenesis of non-small cell lung carcinoma cells [15] and pulmonary hypertension [16], literature is scarce in the fields of asthma and COPD. The increased activity of JMJD2B has been reported in literature to epigenetically regulate endothelial-to-mesenchymal transition (EndMT), an important process in wound healing after tissue injury, where the affected endothelial cells become fibroblast-like, leading to tissue fibrosis [12]. Further, suppression of JMJD2B prevented EndMT-induced reduction of H3K9me3 and subsequent expression of EndMT-related genes. This indicates an important role of JMJD2B in promoting a profibrotic phenotype. In our findings, the elevated expression and activity of JMJD2B at baseline in asthmatic fibroblasts may indicate an aberrant fibroblast phenotype in asthma and ongoing fibrosis even in the absence of active or apparent inflammation clinically. This signifies a potential translational use in the form of early inhibition of the activity of JMJD2B to slow down, or even prevent, fibrosis in the airways of asthmatic individuals.

IL-13 is a key pathogenic player in asthma that is known to induce mononuclear and eosinophilic inflammation, mucus hyperplasia with cellular metaplasia, and fibrosis in the subepithelial layers of the airways, eventually ending up with airway obstruction [17]. In addition to its increased baseline expression in asthmatic fibroblasts, JMJD2B levels were further induced in asthmatic airway fibroblasts upon exposure to IL-13 (Figure 3(a)). IL-13 also augmented the demethylation of histone H3, suggesting a potential mechanism involving JMJD2B in IL-13-mediated activation of fibroblasts which plays an important role in promoting fibrosis as well as inflammatory processes in asthma. It is interesting to note here that our literature search has shown no previous literature demonstrating any direct relation between IL-13 levels and JMJD2B. This is, therefore, one of the first reports indicating the involvement of IL-13-mediated JMJD2B expression in the pathogenesis of asthma.

Despite the well-recognized immunopathobiology of IL-13 in asthma, clinical trials of anti-IL-13 monoclonal antibodies (mAbs) in patients with severe asthma have been largely ineffective. For instance, the STRATOS1 and STRATOS2 phase 3 clinical trials that assessed the safety and efficacy of Tralokinumab for the treatment of severe, uncontrolled asthma did not show significant improvement in asthma outcomes [18]. Further, anothermAb, GSK679586, although well-tolerated, did not demonstrate clinically meaningful improvements in patients with severe asthma [19]. Although JMJD2B expression was induced by IL-13, the elevated baseline expression of this enzyme in asthmatic fibroblasts suggests constitutively high expression of JMJD2B in asthmatic fibroblasts. In this context, JMJD2B may serve as a potential therapeutic target downstream of IL-13. Therefore, targeting JMJD2B may help alleviate IL-13-mediated biological responses in asthma. Our study also showed increased JMJD2B activity in the nucleus of asthmatic fibroblasts compared to their healthy counterparts. This further indicated a link between the nuclear translocation of JMJD2B and asthma pathogenesis. Hypoxic conditions, as seen in the asthmatic airways [20], have previously been reported to induce increased nuclear localization and activity of JMJD2B in pulmonary arterial smooth muscle cells [16]. This study also identified potential biological processes targeted by JMJD, which included proliferation, apoptosis, metabolism, and inflammation, all of which are dysregulated in asthmatic fibroblasts. This further reinforces the pathogenic role of increased JMJD2B activity in promoting airway remodeling.

Increasing evidence suggests an important role of environmental epigenetic regulation in shaping the different asthmatic phenotypes. Importantly, the reversibility of these epigenetic modifications is an attractive feature that may be exploited for the development of novel epigenetic drugs. It would be interesting to explore the use of epigenome-modifying tools in targeting the various hallmark features of asthma, including airway remodeling and airway inflammation.

In an era where the importance of epigenetic alterations in fibrosis and inflammation is increasingly being appreciated, we demonstrate here a novel epigenetic control of fibrosis mediated by JMJD2B in asthmatic airways. Increased activity of histone demethylase JMJD2B is induced by IL-13-mediated profibrotic and proinflammatory conditions.

## Figures and Tables

**Table 1:** DEGs involved in histone trimethylation and or demethylation.

MyList	Description	Biological process (GO)
JMJD2B	Lysine demethylase 4B	GO:0070544 histone H3-K36 demethylation; GO:0033169 histone H3-K9 demethylation; GO:0070076 histone lysine demethylation
JMJD2C	Lysine demethylase 4C	GO:1900113 negative regulation of histone H3-K9 trimethylation; GO:0070544 histone H3-K36 demethylation; GO:1900112 regulation of histone H3-K9 trimethylation
JMJD3	Lysine demethylase 6B	GO:0071557 histone H3-K27 demethylation; GO:0060992 response to fungicide; GO:0070076 histone lysine demethylation
PTDSR	Jumonji domain containing 6, arginine demethylase and lysine hydroxylase	GO:0018395 peptidyl-lysine hydroxylation to 5-hydroxy-L-lysine; GO:0070077 histone arginine demethylation; GO:0070078 histone H3-R2 demethylation

## Data Availability

The datasets used in the present study are available from the corresponding authors upon reasonable request.

## Supplementary Materials

Supplementary Materials Supplementary Figure: original whole blot of Figure 3(c). Western blot analysis showing JMJD2B and trimethylated histones H3 lysine residue at K36 protein levels in the normal and asthmatic fibroblasts upon IL-13 stimulation. Supplementary Table: gene sets that are regulated or affected by histone modification with overlap with the identified DEGs using Enrichr online tool (https://maayanlab.cloud/Enrichr/enrich#) Epigenomics Roadmap HM ChIP-seq. Only sets with adjusted *p* value < 0.05 were selected that were related to fibroblasts.
