# Supplementary material for: IL-13 Augments Histone Demethylase JMJD2B/KDM4B Expression Levels, Activity, and Nuclear Translocation in Airway Fibroblasts in Asthma
Source: J Immunol Res. 2021 Feb 22;2021:6629844. doi: 10.1155/2021/6629844 (PMC7920726; doi:10.1155/2021/6629844)
Supplement: Supplementary Materials — Supplementary Figure: original whole blot of Figure 3(c). Western blot analysis showing JMJD2B and trimethylated histones H3 lysine residue at K36 protein levels in the normal and asthmatic fibroblasts upon IL-13 stimulation. Supplementary Table: gene sets that are regulated or affected by histone modification with overlap with the identified DEGs using Enrichr online tool (https://maayanlab.cloud/Enrichr/enrich#) Epigenomics Roadmap HM ChIP-seq. Only sets with adjusted p value < 0.05 were selected that were related to fibroblasts. [file 6629844.f1.zip › Supplementary Description.docx]

**Supplementary Material:**

**Supplementary Figure:** Original whole blot of Fig. 3C. Western bolt analysis showing JMJD2B and trimethylated histones H3 lysine residue at K36 protein levels in the normal and asthmatic fibroblasts upon IL-13 stimulation.

**Supplementary Table**: Gene sets that are regulated or affected by histone modification with overlap with the identified DEGs using Enrichr online tool (https://maayanlab.cloud/Enrichr/enrich#) Epigenomics Roadmap HM ChIP-seq. Only sets with adjusted p-value <0.05 were selected that were related to fibroblasts
