# Supplementary material for: IL-13 Augments Histone Demethylase JMJD2B/KDM4B Expression Levels, Activity, and Nuclear Translocation in Airway Fibroblasts in Asthma
Source: J Immunol Res. 2021 Feb 22;2021:6629844. doi: 10.1155/2021/6629844 (PMC7920726; doi:10.1155/2021/6629844)
Supplement: Supplementary Materials — Supplementary Figure: original whole blot of Figure 3(c). Western blot analysis showing JMJD2B and trimethylated histones H3 lysine residue at K36 protein levels in the normal and asthmatic fibroblasts upon IL-13 stimulation. Supplementary Table: gene sets that are regulated or affected by histone modification with overlap with the identified DEGs using Enrichr online tool (https://maayanlab.cloud/Enrichr/enrich#) Epigenomics Roadmap HM ChIP-seq. Only sets with adjusted p value < 0.05 were selected that were related to fibroblasts. [file 6629844.f1.zip › Supplementary Table.docx]

**Supplementary Table**: Gene sets that are regulated or affected by histone modification with overlap with the identified DEGs using Enrichr online tool (https://maayanlab.cloud/Enrichr/enrich#) Epigenomics Roadmap HM ChIP-seq. Only sets with adjusted p-value <0.05 were selected that were related to fibroblasts

| Term | Overlap | Adjusted P-value | Combined Score | Genes |  |
| --- | --- | --- | --- | --- | --- |
| H2BK20ac IMR90 | 114 | 6.08E-09 | 1.895094 | 35.85159 | OXTR; NAB1; TNC; SMC5; C14ORF93; SLC4A4; SLC8A1; CDH6; DOCK10; MTRF1; SALL1; CREB3L1; CHEK1; PLCE1; C9ORF150; PITX1; TBC1D22B; IL13RA1; PDK1; SOX5; TMOD1; MAGI1; MYLIP; C5ORF13; PRKCI; IGFBP5; PSG1; ADAM19; PSG9; PSG5; PAPPA; PSG6; HIST1H2BG; IL6ST; ALPK1; KHDRBS1; GMPR; AK5; LPP; PDGFD; PIP5K1A; WNT2; WWTR1; ATP8B4; CPSF6; ABCA6; FBXO38; PARVA; ZFHX4; PBX1; FOSL2; FEN1; FASTKD2; WFDC1; SLC2A3; ALAD; TMEM100; B3GALT2; LRRTM2; PHACTR2; AOX1; IKBKG; DST; MME; BTN3A1; ITGA3; MMP3; RANGAP1; SERPINB8; HIST2H2BE; ASPN; CLDN11; SLC7A5; CDCP1; MDFIC; ITGA7; ANGPTL4; BCAT1; MET; FJX1; CUEDC1; PRKAA1; SEMA3C; SEMA3B; HSD17B6; TFPI; ARNTL2; KIAA0754; GNA13; KCTD20; PALMD; ABI3BP; HAS2; APBB2; TBL1X; PDLIM5; GPC6; LDLR; CA12; GALNT6; NQO1; GALNT3; TRPA1; ATP2B4; PDE4DIP; PLEKHA4; CFLAR; PTPN14; TTLL7; KLF7; DLC1; FLJ43663; KLF9; SNTB1 |
| H2BK12ac IMR90 | 258 | 3.31E-09 | 1.551716 | 30.29735 | OXTR; SMC5; SMC3; SLC4A4; ANTXR1; AQP3; CRKL; GLS; SMC2; EDNRA; ANPEP; CHEK1; DPF3; IL12A; C9ORF150; PELO; PDK1; SOX5; C5ORF13; PSG1; FNDC3A; ARMC8; HSPG2; SIGIRR; EML4; CFDP1; EWSR1; ZC3H11A; RUFY3; PAPPA; KIAA0368; PSG6; KDELR3; ALPK1; SLC35D1; RRAD; GATA6; C6ORF97; ZBTB1; STC1; TAPBPL; APH1B; PDGFD; PIP5K1A; ST3GAL5; MAP4; ATP6V1C1; HIST1H3D; GSTM4; HEXIM1; WWTR1; JAG1; DMPK; TNFSF15; RDX; BRAF; PARVA; PBX1; FLI1; PAFAH2; FOSL2; TPX2; PPFIBP1; ANP32E; NMT2; ATM; APOBEC3B; DHRS12; NRP1; FEN1; FASTKD2; CLTC; GPR20; WFDC1; CHP; PCSK5; EDA2R; EEA1; PPP3CB; C1QTNF1; ZMYM2; B3GALT2; IKBKG; JAK2; SERPINB1; PARP3; MME; BTN3A1; SPHK1; NCOA3; TCF12; BGN; FLJ10038; PAWR; NSUN6; SERPINB8; SLC7A6; CDCP1; MDFIC; MTF2; FJX1; VAMP2; RABGAP1; TANK; NAPG; ARNTL2; KIAA0754; RELN; APOL6; SNX27; HAS2; APBB2; TBL1X; GPC6; CA12; NQO1; MAP3K1; GK; PLEKHA1; PKM2; WWP2; PLEKHA4; HIPK1; SULF1; HIPK3; TTLL7; FABP3; DLC1; CENPN; RAB3B; ZWILCH; NAB1; HFE; NAB2; TNC; IRS2; NUCKS1; SLC8A1; DOCK10; MTRF1; IPO8; GRB10; ENPP1; PITX1; IL6R; IL13RA1; TMOD1; MAGI1; MYLIP; CARD10; LOC339524; POSTN; CEP135; IGFBP5; FBXW11; SWAP70; ADAM19; ADNP; IL6ST; SCG2; GRIA1; FBN2; KHDRBS1; COPA; PXN; ARHGAP19; GMPR; LIN7A; HMMR; LPP; ACAT2; NPAS2; SOCS3; VTN; SBNO2; BAG4; HRH1; ATXN7; PGK1; SOCS6; SLC15A3; WNT2; SLC38A4; MCL1; ATP8B4; HPS1; DHCR24; KLHL24; TNFRSF10D; ISG20; DLG1; CPD; MDM2; NF1; ALPL; LRP12; PHTF2; DDX3X; ATP2A2; SLC2A3; CHD3; SENP6; TMEM100; FLCN; C6ORF106; PSD3; DBT; LRRTM2; PHACTR2; HIST1H1C; KLF12; TGIF2; DST; ITGA3; DDX58; ACTR5; MMP3; PLA2G4C; ETV1; RANGAP1; ETV5; ASPN; TGFBR1; TGFBR2; RBL2; ADORA2B; RASA2; BMP2K; ITGA7; ACPP; MET; CUEDC1; NFAT5; PRKAA1; SEMA3B; FGL2; TFPI; GNA13; CCL7; KCTD20; PALMD; IGF2BP3; POLR2L; SPAG9; GALNT6; RRM2; TRPA1; EYA2; PCDH7; ATP2B4; TPD52L1; PTPN14; SCD; KLF9; FLJ43663; SEC24D; KCNK2; SNTB1 |
| H2BK120ac IMR90 | 234 | 2.44E-08 | 1.537331 | 26.94708 | PGAP1; SMC5; SMC3; ANTXR1; SMC2; CDC20; EDNRA; CHEK1; PLCE1; IL12A; ATXN7L1; LGALS8; PELO; TMPO; TRIM23; SOX5; DDX17; PSG1; ARMC8; EML4; CFDP1; EWSR1; ZC3H11A; RUFY3; KIAA0368; PSG6; TRIB3; KDELR3; ALPK1; TRIB2; ZBTB1; TAPBPL; APH1B; PDGFD; PIP5K1A; MAP4; HIST1H3D; HEXIM1; FZD1; WWTR1; PLK3; JAG1; DMPK; RDX; PARVA; HSPBAP1; PBX1; PAFAH2; FOSL2; NFATC4; MSRA; EHD2; PPFIBP1; ID2; FUBP1; ANP32E; NMT2; ATM; APOBEC3B; HBS1L; NRP2; FEN1; PHF20; FASTKD2; CLTC; CHP; EDA2R; B3GALT2; IKBKG; JAK2; GABPA; RALGPS2; TEAD4; EDN1; MME; BTN3A1; NCOA3; TCF12; FLJ10038; PAWR; CACYBP; GFRA1; SERPINB8; HIST2H2BE; CLDN11; CDCP1; MDFIC; VAMP2; TLR3; KIAA0754; RELN; APOL6; ABI3BP; PGGT1B; HAS2; APBB2; CD58; GPC6; LDLR; CBR3; CA12; NQO1; SVIL; MON2; MAP3K1; WWP2; PLEKHA4; HIPK1; DHRS3; HIPK3; ZNF33B; TTLL7; CENPF; CENPN; F2RL2; ZWILCH; NAB1; HFE; NUCKS1; SLC8A1; THG1L; DOCK10; MTRF1; IPO8; FLJ13197; FPGS; PIM1; GRB10; ENPP1; IL13RA2; IL6R; FADS1; IL13RA1; MYLIP; KIAA0090; CEP135; IGFBP5; FBXW11; SWAP70; USP3; TBC1D2B; ADAM19; GPD2; PLAA; ADNP; HIST1H2BG; IL6ST; STAM2; SLC26A2; PXN; GMPR; LIN7A; SLC1A4; HMMR; AK5; LPP; NPAS2; FUT4; ATXN7; STAP2; SLC15A3; WNT2; SKIL; SLC38A4; MCL1; SMAD1; SMAD3; FBXO38; SYNJ2; TNFRSF10D; ISG20; AHI1; ALS2CR8; MDM2; NF1; ALPL; NF2; PHTF2; DDX3X; ITGB3; ATP2A2; SLC2A3; CHD3; SENP6; FLCN; C6ORF106; PSD3; LRRTM2; AOX1; CASP2; ME3; TGIF2; TRPC6; DST; TRPC3; ITGA3; DDX58; MMP3; PLA2G4C; PAPD4; ETV1; QRSL1; RANGAP1; ASPN; TGFBR1; TGFBR2; RBL2; ITGA7; ACPP; SOS1; MET; RBMS2; NFAT5; PRKAA1; CEBPG; FGL2; GNA13; RMND5A; KCTD20; TAF1A; FAM63B; IGF2BP3; SPAG9; GALNT6; RRM2; TRPA1; PCDH7; ATP2B4; PDE4DIP; CFLAR; CDC42BPA; KLF9; FLJ43663; SNTB1 |
| H2BK15ac IMR90 | 270 | 1.27E-06 | 1.414152 | 19.19902 | NCKAP1; OXTR; PGAP1; SMC5; ENO2; SMC3; SLC4A4; AQP3; ICAM1; SMC2; EDNRA; CREB3L1; CHEK1; IL12A; ATXN7L1; LGALS8; PELO; TBC1D22B; TRIM23; SOX5; C5ORF13; PRKCI; MBNL2; IL1R1; CTPS; PSG9; INPP4B; CFDP1; RUFY3; PAPPA; TFAM; KDELR3; ALPK1; ATP5S; RRAD; SDC2; ZBTB1; TAPBPL; ZC3HAV1; APH1B; DYM; ST3GAL5; HIVEP2; MAP4; ATP6V1C1; GSTM4; HEXIM1; FZD1; WWTR1; PLK3; YES1; APOBEC3G; DMPK; IFI44; CDC7; HSPBAP1; PHC3; FLI1; FOSL2; NFATC4; EXT1; ID2; FUBP1; NMT2; ID3; ATM; APOBEC3B; DHRS12; FEN1; FASTKD2; CLTC; GPR20; WFDC1; CHP; IDE; WASL; PCSK5; EDA2R; B3GALT2; TCP11L1; IDS; IKBKG; JAK2; GABPA; TEAD4; BTN3A1; SPHK1; NCOA3; TCF12; BGN; CACYBP; AMPD3; SERPINB8; HIST2H2BE; CLDN11; SLC7A5; CDCP1; MDFIC; MTF2; GAS1; ANGPTL4; VAMP2; RABGAP1; HSD17B6; EGFR; NAPG; ARNTL2; FSTL3; KIAA0754; APOL6; SNX27; ABI3BP; PGGT1B; PCNX; PLAGL2; APBB2; TBL1X; MAP4K5; GPC6; CBR3; NQO1; MAP3K1; GK; PLEKHA1; PKM2; G0S2; PLEKHA4; HIPK3; DCBLD2; FABP3; PKN2; ZWILCH; NAB1; C11ORF71; NUCKS1; MANEA; SLC8A1; THG1L; CDH6; DOCK10; MTRF1; IPO8; SALL1; ADORA1; PIM1; ENPP2; ENPP1; SIRPA; IL13RA2; KPNA1; IL13RA1; POSTN; CEP135; FBXW11; SWAP70; USP3; HGF; ADAM19; ALDH3A2; GPD2; PPIG; ADNP; IL6ST; PPIA; STAM2; KHDRBS1; COPA; RBM8A; ABHD2; PXN; RHOBTB3; GMPR; LIN7A; SLC1A4; HMMR; LPP; NPAS2; NUP160; FBXO42; VTN; BAG4; HRH1; PGK1; MSC; WNT2; SLC38A4; MCL1; ATP8B4; CPSF6; DHCR24; SYNJ2; ISG20; NR4A2; AHI1; CNOT2; NFE2L3; NF1; LRP12; PRR5; RERE; PHTF2; FLT1; DDX3X; LOC282997; ITGB3; HTR2B; C10ORF28; SENP6; TMEM100; SENP7; C6ORF106; SNAPC3; PSD3; LRRTM2; CASP2; ME3; MAN1A1; LRRFIP1; SCYL2; HIST1H1C; KLF12; TRPC6; DST; DDX58; TRPC1; ETV1; RANGAP1; ASPN; TGFBR1; RBL2; DNAJC7; ADORA2B; NUP50; RASA2; BMP2K; BIRC5; ITGA7; ACPP; BCAT1; MET; SSX2IP; SOS2; PRPS1; NFAT5; PRKAA1; SEMA3B; FGL2; HMGCR; TFPI; PTPLA; KCTD20; PALMD; FAM63B; IGF2BP3; CLASP2; SPAG9; GALNT6; TTC12; MYO10; TRPA1; ATP2B4; CFLAR; GALC; SCD; ST7; KLF9; FLJ43663; SEC24D; MBTPS2; SNTB1 |
| H3K9ac IMR90 | 715 | 9.29E-04 | 1.193402 | 8.331959 | RB1; CYFIP2; ATF2; NCKAP1; PGAP1; ZNF174; POP1; LOC389634; TRRAP; SMC5; TMEM97; EPRS; SMC3; SLC4A4; BACH2; SMC2; SCAMP4; NSDHL; DPYSL4; CREB3L1; PLCE1; HOXA7; C5ORF13; STARD5; ACOT7; CACNA2D1; PRKCE; NGDN; HUS1; ARMC8; SCAMP1; ZNF14; SIGIRR; CH25H; PRKAR1B; ZC3H11A; RUFY3; GPR161; PARD3; KIAA0368; AGTR1; MTMR1; C6ORF97; ENDOD1; TAPBPL; ZNF24; KIAA1009; PDGFD; MYO6; HMOX1; EDEM3; GSTM4; FZD1; B3GALNT1; PLK3; ABCA6; EIF2AK1; IRF2BP1; CDC7; BRAF; CDC6; PHC3; FLI1; PAFAH2; ELL3; FOSL2; NFATC4; PCDHGA10; PPFIBP1; PPFIBP2; SLC6A9; C19ORF42; FASN; SSPN; NMT2; CDK10; DCHS1; HBP1; EZH2; ARF3; C9ORF91; SMG1; SETD2; PHF20; SETD6; DENND1A; QPRT; CLTC; ARHGAP1; ARHGAP6; EEA1; C16ORF72; C1QTNF1; CASP10; B3GALT2; ENSA; TCP11L1; PBK; IKBKG; JAK2; TIMP4; CEP55; TEAD4; DFFB; TCF12; BGN; PAWR; C6ORF62; SERPINB8; HIST2H2BE; TMEFF1; SLC7A6; VAMP1; GAS1; COL21A1; ANGPTL4; SLC29A1; GART; GAS7; IDUA; DOM3Z; SLC7A1; EGFR; GGA2; LMAN1; BCLAF1; APOL6; ABI3BP; UCP2; STX6; SLC17A5; PLAGL2; HAS2; ZNF225; TAF9B; ZNF224; YKT6; GK; STAT2; ATAD2; SAMD4A; G0S2; HIPK1; SULF1; DHRS3; ZNF33B; TTLL7; FABP3; CENPF; TTLL5; SNX16; TAOK1; NFIC; PKN2; CENPN; CENPQ; SOLH; CDKN3; KCNG1; RAB3B; PECR; CCNT2; CLSTN3; NAB1; C11ORF71; RORA; IRS2; FRY; SLC8A1; THG1L; EDC3; MTRF1; IPO8; SALL1; FGF9; SLC39A9; C15ORF5; ADORA1; PIM1; LEPR; KPNA5; MAP3K6; GYG2; KPNA1; IL13RA1; TLE4; MAGI1; MYLIP; FAM48A; TLE2; CEP135; SWAP70; USP3; VASH2; CHD1L; POLRMT; PIAS1; SMO; DMWD; TCTA; TNIK; ADNP; IL6ST; GOLGA8A; CSNK1G1; ZNF430; GRIA1; FTO; ZNF551; KHDRBS1; SLC26A2; CBFB; SMYD5; INSIG1; ITPR1; GMPR; LIN7A; ABHD6; HMMR; AK5; LIN7B; RLF; ACAT2; NPAS2; HSP90B1; FBXO42; PORCN; BAG4; ATXN7; CLTCL1; TPR; G3BP2; PGK1; PLXNA2; EIF4EBP2; AP4S1; SOCS6; RAB6A; SLC15A3; SLC38A4; NEK9; ANKRD26; ATP8B4; FBXO38; HPS1; TNFRSF10C; CNOT4; ALS2CR8; GNL3L; MAFB; GGCX; CPD; CNOT2; CNOT3; REEP2; NFE2L3; MDM2; PDCL; NF1; PIN4; TUBGCP3; DHCR7; BCL2L1; SNAP25; DIDO1; FLT1; ITGB3; PIK3CD; FOXM1; SYNE2; C10ORF28; TRO; GM2A; SGCB; C6ORF106; SCRN3; PSD3; WAPAL; NEO1; CEP290; IDH3A; RFC3; TRPC6; ITGA4; HMGCS1; TRPC3; ACTR5; TRPC1; ATRX; VPS13D; LANCL1; ELAC1; VPS13A; PAPD4; RANGAP1; ASPM; PIK3CA; RPL37A; BMP2K; LANCL2; CCDC41; BIRC5; ITGA6; ANG; ACPP; MET; DTL; ZNF510; SOS2; PRPS1; NFAT5; RNF32; PRKAA1; ROCK1; SEMA3C; ZBTB48; SEMA3B; CEBPG; FGL2; ZBTB43; LRP4; TTK; HDAC9; PTPLA; KCTD20; IGF2BP3; LRRC8B; ZNF500; ATP9B; CNNM3; POLR2L; SLC35A2; SPAG9; RNGTT; TRPA1; PCDH7; CEP152; HIP1R; CFLAR; PTPN12; TPD52L1; BAIAP2; TM6SF1; PTPN14; BICC1; MYO1D; TSPAN13; APC; NOVA1; PRC1; MAB21L1; FLJ43663; TRIM33; GPR172A; MT1M; IFI30; C14ORF93; NUDT6; ANTXR1; AQP3; ICAM1; IKZF5; CCT6B; GOLGA4; EDNRA; RPS6KA5; CHEK1; DPF3; IL12A; ATXN7L1; LGALS8; SKP2; PTGDS; TRIM21; TBC1D22B; TMPO; TRIM23; PDK1; SOX5; ARSA; AQR; ATP6V1G2; SFMBT1; METTL1; ACSL1; FLT3LG; CTPS; INPP4B; CFDP1; BTD; C10ORF118; GUF1; TFAM; COL8A1; LPPR4; FAM64A; KDELR3; KIF20A; ALPK1; TP53; OBSL1; ATP5S; PTGFR; TMEM63A; MAOA; ZBTB1; BRCC3; ZBTB3; TTBK2; MBTD1; SYNGR2; APH1B; SPAST; TSPAN9; DHX35; DYM; ASL; APOE; HIVEP2; MAP4; STXBP6; ARSD; ATP6V1C1; ZBED4; SPTBN1; PCK2; WWTR1; EGR3; APOBEC3F; YES1; APOBEC3G; GADD45B; SLC35E2; TNFSF15; IFI44; PILRB; PARVA; HSPBAP1; SMARCA2; NET1; POLA1; MSRA; TPX2; LOC81691; WNK1; ID2; FUBP1; RANBP10; ANP32E; ID3; HIST1H4H; ATM; APOBEC3B; DHRS12; NRP1; HBS1L; ZCCHC11; MLF1IP; FASTKD2; CTSZ; RSF1; WASL; MLL; PCSK5; LIMD2; PPP3CB; AKAP11; SPTLC2; C14ORF1; RSRC1; ZMYM6; KIF13A; IDS; AASS; GABPA; HTATIP2; SREBF1; NCOA2; PARP3; EDN1; MME; BTN3A1; NCOA3; MST1; GAB1; C17ORF80; FLJ10038; CACYBP; KIF23; MRPS6; ZFX; CTTNBP2NL; C9ORF9; CTTN; NOV; C9ORF5; CENPC1; ARHGEF3; ALDOC; ARHGEF7; MATN3; TLR3; DNASE1L1; FAM59A; PTGER4; SP100; RABGAP1; STX16; HSD17B6; NAPG; ARNTL2; ARNTL; KIAA0754; EXOSC5; RELN; GPC1; HSF2; HSF1; PGGT1B; CTNNA1; APBB2; MSX1; CD58; GINS1; NQO1; SVIL; GINS2; MON2; GCH1; PLEKHA1; YIPF2; NFYA; VEGFB; PEX10; RAB11B; BOP1; RSL1D1; GLB1L; MARS; FKBP1B; TRIP13; F2RL2; MAD2L1; ZFAND6; HFE; PRDM2; UBE3B; MANEA; DOCK10; RGS5; FLJ13197; ENPP2; NUSAP1; ENPP1; SIRPA; GGT1; TMEM38B; CD97; GUCY1A3; CARD10; KIAA0090; MSX2; LIG1; FBXW11; LIG4; LMO7; PDGFRL; ADAM19; ALDH3A2; ADAM15; DOK5; GPD2; MASP1; PPIG; HIST1H2BG; STAM2; FBN2; DDX6; IDI1; COPA; RBM8A; YTHDC2; SEL1L; SLC1A1; PSEN2; ARHGAP19; SLC1A4; CACNA1C; FUT4; NUP160; EFNB2; DPP4; ARHGAP22; CCNB2; EBP; CCNB1; HRH1; COX11; TASP1; KIF3C; LONRF1; WNT2; MYH10; SMAD1; SMAD4; CPSF6; SYT1; VDR; HSPA4; SMURF1; RARRES1; GGH; KLHL24; SMAD5; BMP6; SUZ12P; ANKHD1; ISG20; NR4A2; AHI1; NR4A1; IL6; BMP2; CPS1; DLG5; PHF14; ALPL; LSM14B; BRWD1; ZCCHC4; ZCCHC2; LRP12; PRR5; DOCK6; SNED1; PHTF2; DDX3X; DYRK2; RND2; SLC2A6; SLC2A8; SEC61A2; SENP6; TMEM100; FLCN; SENP7; SNAPC2; SNAPC3; DBT; LRRTM2; AOX1; CASP2; PDE4A; MAN1A1; MYBL1; ABCD1; RBM5; SCYL2; SNTA1; HIST1H1C; ARHGEF11; KLF12; TGIF2; PTGIS; DDX58; DCAKD; PLA2G4C; SHROOM2; ETV1; PPP2R5C; QRSL1; LSS; ETV5; TGFBR1; TGFBR2; RBL2; SETBP1; EEF1D; RASA2; MVD; RBKS; DCTN5; MVK; RNF6; PRELP; HMGCR; NR2C1; AURKA; RMND5A; PALMD; TDG; TAF1A; FAM63B; PDLIM5; CLASP2; REPS1; ATF7IP; GALNT6; TTC12; RRM2; C4ORF29; USP20; EYA2; ZNF180; PDE4DIP; MAPK14; SHCBP1; C14ORF135; CLCN7; GALC; GALE; SCD; LPIN1; MBTPS2; OTUD3 |
| H4K8ac IMR90 | 747 | 0.003918 | 1.163819 | 6.449956 | RB1; ATF2; NCKAP1; PGAP1; ZNF174; POP1; TRRAP; SMC5; TMEM97; EPRS; SMC3; SLC4A4; BACH1; GLS; SMC2; SCAMP4; NSDHL; AKT2; HOXA7; DDX17; C5ORF13; ACOT7; CACNA2D1; PRKCE; NGDN; HUS1; SOX11; FNDC3A; ARMC8; SCAMP1; ZNF14; EML4; CH25H; MTAP; PRKAR1B; EWSR1; BIN1; RUFY3; GPR161; ZNF395; C6ORF97; ENDOD1; TAPBPL; ZC3HAV1; ZNF24; KIAA1009; PDGFD; MYO6; PIP5K1A; EDEM3; ABCA1; GSTM4; HEXIM1; FZD1; B3GALNT1; PLK3; DMPK; ABCA6; EIF2AK1; RDX; IRF2BP1; CDC7; BRAF; CDC6; PBX1; FLI1; PAFAH2; ELL3; FOSL2; NFATC4; PCDHGA10; SQLE; PPFIBP1; PCDHGA12; PPFIBP2; SLC6A9; C19ORF42; FASN; SSPN; NMT2; CDK10; DCHS1; HBP1; EZH2; ARF3; CDKN1C; C9ORF91; SMG1; SETD2; PHF20; PHF1; ELN; SETD6; DENND1A; QPRT; CLTC; ARHGAP1; GPR20; CHP; C16ORF72; C1QTNF1; CASP10; ENSA; TCP11L1; PBK; IKBKG; TK1; JAK2; TIMP4; CEP55; TEAD4; DFFB; DUSP3; SPHK1; TCF12; BGN; PAWR; ELOVL6; NSUN6; C6ORF62; SERPINB9; SERPINB8; HIST2H2BE; SLC7A5; SLC7A6; MTF2; VAMP1; GAS1; ANGPTL4; SLC29A1; ZNF236; GART; VAMP2; GAS7; NFIX; IDUA; DOM3Z; SLC7A1; TANK; FSTL3; GGA2; LMAN1; BCLAF1; APOL6; SNX27; ABI3BP; UCP2; STX6; RPL14; SLC17A5; PLAGL2; HAS2; ZNF225; TAF9B; ZNF224; YKT6; NKTR; GK; STAT2; G0S2; WWP2; HIPK1; SULF1; DHRS3; HIPK3; DCBLD2; ZNF33B; TTLL7; FABP3; CENPF; TTLL5; SNX16; TAOK1; NFIC; PKN2; CENPN; CENPQ; SOLH; CDKN3; PECR; CCNT2; CLSTN3; NAB1; PDCD6; C11ORF71; TNC; IRS2; SLC8A1; IPO4; TM7SF2; THG1L; EDC3; MTRF1; IPO8; FGF9; SLC39A9; C15ORF5; ADORA1; PIM1; LEPR; KPNA6; GRB10; KPNA5; MAP3K6; PITX1; FADS1; C8ORF44; KPNA1; IL13RA1; TLE4; MAGI1; MYLIP; FAM48A; CEP135; SWAP70; USP3; UBE2E1; FRMD4A; CHD1L; POLRMT; PIAS1; SMO; TCTA; TNIK; ADNP; IL6ST; UTRN; CSNK1G1; ZNF430; FTO; ZNF551; KHDRBS1; SLC26A2; CBFB; SMYD5; INSIG1; ABHD2; MAZ; ITPR1; GMPR; LIN7A; ABHD6; HMMR; AK5; LIN7B; RLF; ACAT2; NPAS2; HSP90B1; FBXO42; VTN; SBNO2; PORCN; ATXN7; CLTCL1; TPR; G3BP2; PGK1; AP4S1; RAB6A; SLC15A3; SKIL; SLC38A4; MCL1; NEK9; ANKRD26; ATP8B4; HPS1; TNFRSF10C; ZBTB10; DHCR24; SYNJ2; C1ORF63; CNOT4; ALS2CR8; GNL3L; MAFB; GGCX; NACA; CPD; CNOT2; REEP2; NFE2L3; MDM2; PDCL; NF1; PIN4; TUBGCP3; DHCR7; NF2; SLC26A6; FLT1; LOC282997; HTR2B; FOXM1; C10ORF28; ALAD; TRO; HHEX; GM2A; SGCB; C6ORF106; SCRN3; PSD3; WAPAL; ME3; NEO1; EDIL3; CEP290; IDH3A; RFC3; TRPC6; ITGA4; DST; HMGCS1; TRPC3; ITGA3; ACTR5; TRPC1; ATRX; VPS13D; LANCL1; ELAC1; VPS13A; PAPD4; RANGAP1; RAD23B; PIK3CA; DNAJC7; ADORA2B; RPL37A; BMP2K; LANCL2; CCDC41; BIRC5; ITGA7; ANG; ACPP; SOS1; MET; DTL; SSX2IP; ZNF510; SOS2; PRPS1; NFAT5; RNF32; PRKAA1; ROCK1; SEMA3C; CEBPD; ZBTB48; SEMA3B; FGL2; ZBTB43; LRP5; TTK; PTPLA; KCTD20; IGF2BP3; LRRC8B; ZNF500; ATP9B; CNNM3; SLC35A2; SPAG9; RNGTT; TRPA1; PCDH7; CEP152; CFLAR; PTPN12; TM6SF1; CDC42BPA; PTPN14; BICC1; DBNDD1; TSPAN13; KIAA0101; APC; PRC1; ST7; FLJ43663; SEC24D; TRIM33; OXTR; GPR172A; IFI30; NUDT6; ANTXR1; ICAM1; CRKL; IKZF5; CCT6B; CDC20; GOLGA4; EDNRA; CHEK1; DPF3; IL12A; ATXN7L1; C9ORF150; PTGDS; TRIM21; PELO; TBC1D22B; TMPO; TRIM23; PDK1; SOX5; ARSA; AQR; ATP6V1G2; METTL1; NPFF; PSG1; FLT3LG; INPP4B; CFDP1; BTD; PSG4; PAPPA; PSG6; C10ORF118; GUF1; COL8A2; TFAM; FAM64A; KDELR3; KIF20A; ALPK1; TRIB2; TP53; OBSL1; ATP5S; PTGFR; TMEM63A; SLC35D1; DHX9; MAOA; ZBTB1; BRCC3; ZBTB3; TTBK2; MBTD1; SYNGR2; APH1B; SPAST; TSPAN9; DHX35; DYM; ASL; HIVEP2; MAP4; STXBP6; ARSD; ATP6V1C1; ZBED4; PCK2; WWTR1; EGR3; APOBEC3F; YES1; APOBEC3G; IFI44; PILRB; HSPBAP1; POLA1; MSRA; TPX2; LOC81691; WNK1; ID2; FUBP1; RANBP10; ANP32E; ID3; HIST1H4H; ATM; APOBEC3B; DHRS12; TOP2A; NRP1; HBS1L; FEN1; ZCCHC11; FASTKD2; WFDC1; RSF1; KIF11; WASL; MLL; PCSK5; LIMD2; PPP3CB; ZMYM2; SPTLC2; C14ORF1; RSRC1; MAP1LC3C; ZMYM6; IDS; DFNB31; AASS; GABPA; HTATIP2; RALGPS2; SREBF1; NCOA2; PARP3; EDN1; MME; BTN3A1; PRMT2; NCOA3; MST1; GAB1; FLJ10038; KIF23; MRPS6; ZFX; CLDN11; C9ORF9; CDCP1; CTTN; MDFIC; C9ORF5; CENPC1; ARHGEF3; ALDOC; MATN3; TLR3; DNASE1L1; PTGER4; RABGAP1; STX16; HSD17B6; NAPG; ARNTL2; ARNTL; KIAA0754; EXOSC5; RELN; GPC1; HSF2; HSF1; PGGT1B; PCNX; CTNNA1; C19ORF6; APBB2; MSX1; CD58; GPC6; C22ORF29; CBR3; CA12; GINS1; NQO1; SVIL; GINS2; MON2; MAP3K1; PLEKHA1; YIPF2; NFYA; VEGFB; PEX10; RAB11B; BOP1; RSL1D1; SPCS3; KITLG; KLHL7; MARS; DLC1; FKBP1B; TRIP13; F2RL2; MAD2L1; ZWILCH; HFE; BUB1B; PRDM2; NUCKS1; MANEA; DOCK10; RGS5; FLJ13197; FPGS; ENPP2; NUSAP1; ENPP1; BAALC; TMEM38B; CD97; CARD10; KIAA0090; POSTN; MSX2; LIG1; FBXW11; HGF; LIG4; LMO7; ADAM19; ALDH3A2; ADAM15; GPD2; PLAA; PPIG; HIST1H2BG; PPIA; STAM2; FBN2; DDX6; IDI1; COPA; RBM8A; YTHDC2; SEL1L; SLC1A1; PSEN2; ARHGAP19; SLC1A4; CACNA1C; LPP; FUT4; NUP160; ARHGAP22; CCNB2; EBP; CCNB1; HRH1; COX11; TASP1; KIF3C; MSC; LONRF1; WNT2; MYH10; SMAD1; MGEA5; SMAD4; CPSF6; SYT1; HSPA4; RARRES1; GGH; KLHL24; SMAD5; ANKHD1; ISG20; AHI1; NR4A1; BMP2; DLG1; NASP; CPS1; DLG5; TRPV4; PHF14; ALPL; LSM14B; BRWD1; ZCCHC4; LRP12; RERE; DOCK6; PHTF2; DDX3X; DYRK2; ATP2A2; SLC2A3; CHD3; SLC2A6; GPHN; HK2; SEC61A2; SENP6; TMEM100; FLCN; SENP7; DBP; SNAPC3; DBT; LRRTM2; AOX1; CASP2; PDE4A; MAN1A1; LRRFIP1; MYBL1; ABCD1; RBM5; SCYL2; SNTA1; ARHGEF11; KLF12; TGIF2; MMP1; DDX58; DCAKD; PLA2G4C; ETV1; QRSL1; ETV5; TGFBR1; TGFBR2; RBL2; EEF1D; MVD; ZP3; CUEDC1; RBMS2; DCTN5; MVK; RNF6; HMGCR; NR2C1; AURKA; RMND5A; PALMD; TAF1A; FAM63B; CLASP2; REPS1; ATF7IP; SPEN; GALNT6; TTC12; RRM2; C4ORF29; USP20; EYA2; ATP2B4; ZNF180; MAPK14; SHCBP1; CLCN7; GALC; GALE; SCD; LPIN1; MBTPS2; SNTB1; OTUD3 |
| H3K18ac IMR90 | 538 | 0.009087 | 1.148123 | 5.397276 | RB1; ATF2; NCKAP1; PGAP1; POP1; TRRAP; EPRS; SMC3; AKT2; HOXA7; C5ORF13; CACNA2D1; PRKCE; HUS1; FNDC3A; ARMC8; SCAMP1; ZNF14; CH25H; MTAP; EWSR1; BIN1; ZC3H11A; RUFY3; GPR161; PARD3; KIAA0368; ZNF395; C6ORF97; TAPBPL; ZC3HAV1; ZNF24; KIAA1009; C7; MYO6; ABCA1; GSTM4; HEXIM1; FZD1; B3GALNT1; PLK3; DMPK; EIF2AK1; RDX; IRF2BP1; CDC7; BRAF; CDC6; PHC3; FLI1; PAFAH2; PPFIBP1; SLC6A9; C19ORF42; FASN; NMT2; DCHS1; HBP1; ARF3; C9ORF91; SMG1; SETD2; PHF1; DENND1A; QPRT; CLTC; ARHGAP1; GPR20; CHP; C16ORF72; CASP10; B3GALT2; TCP11L1; PBK; IKBKG; JAK2; CEP55; TEAD4; DFFB; SERPINB1; DUSP3; SPHK1; TCF12; PAWR; NSUN6; SERPINB8; HIST2H2BE; SLC7A5; MTF2; VAMP1; GAS1; ANGPTL4; GART; VAMP2; GAS7; NFIX; COL11A1; DOM3Z; TANK; LMAN1; BCLAF1; APOL6; ABI3BP; SLC17A5; PLAGL2; HAS2; NKTR; SF3A2; GK; STAT2; ATAD2; WWP2; HIPK1; SULF1; DHRS3; DCBLD2; ZNF33B; TTLL7; FABP3; TAOK1; PKN2; CENPN; SOLH; KCNG1; RAB3B; PECR; CCNT2; CLSTN3; NAB1; NAB2; C11ORF71; IRS2; FRY; SLC8A1; THG1L; EDC3; MTRF1; IPO8; SLC39A9; C15ORF5; ADORA1; PIM1; KPNA5; MAP3K6; PITX1; IL13RA1; TLE4; MAGI1; FAM48A; CEP135; S100A2; IGFBP5; SWAP70; UBE2E1; SMO; TCTA; ADNP; IL6ST; CSNK1G1; ZNF430; GRIA1; KHDRBS1; SLC26A2; CBFB; SMYD5; ABHD2; MAZ; ITPR1; GMPR; LIN7A; ABHD6; HMMR; AK5; RLF; ACAT2; NPAS2; HSP90B1; VTN; PORCN; ATXN7; CLTCL1; TPR; G3BP2; PGK1; AP4S1; RAB6A; SLC15A3; SLC38A4; ATP8B4; FBXO38; HPS1; C1ORF63; TNFRSF10D; CNOT4; ALS2CR8; GNL3L; GGCX; NACA; CPD; CNOT2; NFE2L3; MDM2; PDCL; PIN4; TUBGCP3; DHCR7; NF2; SLC26A6; DIDO1; ITGB3; FOXM1; C10ORF28; TRO; HHEX; GM2A; SGCB; C6ORF106; SCRN3; PSD3; NEO1; IDH3A; EIF5A; RFC3; TRPC6; TRPC3; ITGA3; ACTR5; TRPC1; ATRX; LANCL1; ELAC1; VPS13A; PAPD4; RANGAP1; ASPM; PIK3CA; DNAJC7; BMP2K; LANCL2; CCDC41; ITGA7; ANG; PPP1R12B; ACPP; SOS1; MET; DTL; SOS2; PRPS1; NFAT5; RNF32; PRKAA1; SEMA3B; CEBPG; FGL2; ZBTB43; TTK; KCTD20; IGF2BP3; ATP9B; SLC35A2; SPAG9; TRPA1; PCDH7; HIP1R; CFLAR; PTPN12; TM6SF1; CDC42BPA; PTPN14; APC; ST7; KLF9; FLJ43663; SEC24D; KCNK2; GPR172A; C14ORF93; NUDT6; ANTXR1; AQP3; ICAM1; CRKL; CCT6B; CHEK1; IL12A; ATXN7L1; LGALS8; SKP2; PTGDS; TRIM21; TMPO; TRIM23; PDK1; SOX5; ENTPD4; METTL1; IL1R1; PSG1; FLT3LG; CTPS; CFDP1; BTD; PAPPA; C10ORF118; GUF1; TFAM; COL8A1; KDELR3; KIF20A; ALPK1; TP53; OBSL1; ATP5S; TMEM63A; MAOA; ZBTB1; STC1; BRCC3; ZBTB3; MBTD1; APH1B; TSPAN9; ASL; APOE; HIVEP2; STXBP6; ARSD; ATP6V1C1; ZBED4; SPTBN1; PCK2; EGR1; WWTR1; APOBEC3F; YES1; APOBEC3G; TNFSF15; IFI44; HSPBAP1; MSRA; TPX2; LOC81691; ID2; FUBP1; ID3; HIST1H4H; ATM; DHRS12; NRP1; HBS1L; FEN1; ZCCHC11; FASTKD2; WFDC1; RSF1; IDE; KIF11; WASL; MLL; PCSK5; ZMYM2; SPTLC2; RSRC1; ZMYM6; IDS; AASS; GABPA; RALGPS2; NCOA2; PARP3; EDN1; MME; BTN3A1; PRMT2; NCOA3; GAB1; CACYBP; MRPS6; ZFX; CTTNBP2NL; C9ORF9; CTTN; CENPC1; MATN3; TLR3; DNASE1L1; FAM59A; RABGAP1; NAPG; ARNTL2; KIAA0754; RELN; GPC1; MGAT5; PGGT1B; PCNX; CTNNA1; C19ORF6; APBB2; CD58; TBL1X; C22ORF29; CBR3; CA12; NQO1; SVIL; MON2; NFYA; KAZALD1; RAB11B; RSL1D1; SPCS3; KITLG; GLB1L; DLC1; TRIP13; F2RL2; MAD2L1; ZWILCH; HFE; PRDM2; NUCKS1; UBE3B; MANEA; DOCK10; RGS5; FPGS; ENPP2; ENPP1; SIRPA; GUCY1A3; KIAA0090; LIG1; FBXW11; LIG4; LMO7; ADAM19; ALDH3A2; GPD2; PPIG; HIST1H2BG; STAM2; FBN2; DDX6; COPA; RBM8A; YTHDC2; SEL1L; SLC1A1; ARHGAP19; LPP; NUP160; HRH1; COX11; TASP1; STAP2; KIF3C; LONRF1; WNT2; MYH10; SMAD1; MGEA5; SMAD4; CPSF6; HSPA4; KLHL24; SMAD5; SUZ12P; ISG20; NR4A2; AHI1; CPS1; DLG5; BRWD1; LRP12; DOCK6; PHTF2; DDX3X; DYRK2; SLC2A3; SEC61A2; SENP6; TMEM100; FLCN; SENP7; SNAPC3; DBT; LRRTM2; AOX1; CASP2; MAN1A1; ABCD1; SCYL2; HIST1H1C; ARHGEF11; TGIF2; MMP1; DDX58; DCAKD; PLA2G4C; ETV1; PPP2R5C; QRSL1; LSS; ETV5; TGFBR1; RBL2; SETBP1; EEF1D; RASA2; DCTN5; RNF6; HMGCR; NR2C1; AURKA; RMND5A; PALMD; TAF1A; FAM63B; CLASP2; REPS1; ATF7IP; GALNT6; TTC12; RRM2; C4ORF29; EYA2; ZNF180; PDE4DIP; MAPK14; C14ORF135; CLCN7; GALC; REST; SCD; MBTPS2; SNTB1 |
| H3K27ac IMR90 | 755 | 0.020128 | 1.124767 | 4.392945 | RB1; CYFIP2; ATF2; NCKAP1; PGAP1; ZNF174; POP1; TRRAP; SMC5; TMEM97; EPRS; SMC3; BACH1; BACH2; SMC2; SCAMP4; NSDHL; DPYSL4; AKT2; PLCE1; HOXA7; DDX17; C5ORF13; PRKCI; STARD5; ACOT7; CACNA2D1; PRKCE; NGDN; ARMC9; HUS1; SOX11; FNDC3A; ARMC8; SCAMP1; ZNF14; EML4; CH25H; MTAP; PRKAR1B; EWSR1; BIN1; ZC3H11A; RUFY3; GPR161; PARD3; MTMR1; C6ORF97; ENDOD1; TAPBPL; ZC3HAV1; ZNF24; KIAA1009; C7; PDGFD; MYO6; EDEM3; GSTM4; HEXIM1; B3GALNT1; DMPK; ABCA6; EIF2AK1; RDX; IRF2BP1; CDC7; BRAF; CDC6; PHC3; PBX1; FLI1; PAFAH2; ELL3; PCDHGA10; SQLE; PPFIBP1; PPFIBP2; SLC6A9; C19ORF42; FASN; SSPN; NMT2; CDK10; DCHS1; HBP1; EZH2; ARF3; C9ORF91; SMG1; SETD2; PHF20; ELN; SETD6; DENND1A; QPRT; CLTC; ARHGAP1; GPR20; CHP; EEA1; C16ORF72; B3GALT2; ENSA; TCP11L1; PBK; IKBKG; TK1; JAK2; TIMP4; CEP55; TEAD4; DFFB; DUSP3; SPHK1; TCF12; EMP1; PAWR; NSUN6; C6ORF62; SERPINB9; SERPINB8; HIST2H2BE; SLC7A5; TMEFF1; MTF2; VAMP1; GAS1; ANGPTL4; SLC29A1; ZNF236; GART; VAMP2; GAS7; NFIX; CCL11; IDUA; DOM3Z; SLC7A1; FAM117A; GGA2; LMAN1; BCLAF1; APOL6; UCP2; STX6; SLC17A5; PLAGL2; HAS2; ZNF225; TAF9B; ZNF224; YKT6; GK; STAT2; ATAD2; SAMD4A; G0S2; HIPK1; DHRS3; ZNF33B; TTLL7; FABP3; CENPF; TTLL5; SNX16; TAOK1; NFIC; PKN2; CENPN; CENPQ; SOLH; CDKN3; RAB3B; PECR; CCNT2; CLSTN3; NAB1; C11ORF71; RORA; IRS2; FRY; SLC8A1; TM7SF2; THG1L; EDC3; MTRF1; IPO8; SALL1; FGF9; SLC39A9; C15ORF5; ADORA1; PIM1; LEPR; GRB10; KPNA5; MAP3K6; GYG2; PITX1; C8ORF44; KPNA1; IL13RA1; TLE4; MAGI1; MYLIP; FAM48A; CEP135; S100A2; SWAP70; USP3; UBE2E1; VASH2; CHD1L; POLRMT; PIAS1; SMO; TCTA; TNIK; ADNP; IL6ST; GOLGA8A; CSNK1G1; ZNF430; GRIA1; FTO; ZNF551; KHDRBS1; SLC26A2; CBFB; SMYD5; INSIG1; ITPR1; RHOBTB3; GMPR; LIN7A; ABHD6; HMMR; AK5; LIN7B; RLF; ACAT2; NPAS2; HSP90B1; VTN; SBNO2; PORCN; ATXN7; CLTCL1; TPR; G3BP2; PGK1; PLXNA2; EIF4EBP2; AP4S1; NPTX1; SOCS6; RAB6A; SLC15A3; SKIL; SLC38A4; MCL1; NEK9; ANKRD26; FBXO38; HPS1; ZBTB10; DHCR24; C1ORF63; CNOT4; PTPRE; ALS2CR8; GNL3L; MAFB; GGCX; NACA; CPD; CNOT2; CNOT3; REEP2; MDM2; PDCL; NF1; PIN4; TUBGCP3; DHCR7; SLC26A6; DIDO1; FLT1; ITGB3; PIK3CD; FOXM1; SYNE2; WISP2; C10ORF28; TRO; HHEX; GM2A; SGCB; C6ORF106; SCRN3; PSD3; WAPAL; ME3; NEO1; CEP290; IDH3A; RFC3; TRPC6; ITGA4; HMGCS1; TRPC3; ITGA3; ACTR5; TRPC1; ATRX; VPS13D; LANCL1; ELAC1; VPS13A; PAPD4; RANGAP1; RAD23B; ASPM; PIK3CA; RPL37A; BMP2K; LANCL2; CCDC41; BIRC5; ITGA6; ANG; PPP1R12B; ACPP; MET; DTL; SSX2IP; ZNF510; SOS2; PRPS1; NFAT5; RNF32; PRKAA1; SEMA3C; ZBTB48; SEMA3B; CEBPG; FGL2; ZBTB43; LRP4; TTK; PTPLA; KCTD20; IGF2BP3; LRRC8B; ZNF500; ATP9B; CNNM3; POLR2L; SLC35A2; SPAG9; RNGTT; TRPA1; PCDH7; CEP152; HIP1R; CFLAR; PTPN12; TPD52L1; BAIAP2; TM6SF1; CDC42BPA; BICC1; TSPAN13; APC; NOVA1; PRC1; ST7; FLJ43663; SEC24D; TRIM33; GPR172A; IFI30; C14ORF93; NUDT6; ANTXR1; ICAM1; CRKL; IKZF5; CCT6B; CDC20; EDNRA; CHEK1; DPF3; IL12A; ATXN7L1; C9ORF150; LGALS8; SKP2; PTGDS; TRIM21; PELO; TBC1D22B; TMPO; TRIM23; SOX5; ARSA; AQR; ATP6V1G2; ENTPD4; SFMBT1; METTL1; ACSL1; FLT3LG; CTPS; INPP4B; CFDP1; BTD; PSG6; C10ORF118; GUF1; TFAM; COL8A1; LPPR4; FAM64A; KDELR3; KIF20A; ALPK1; TRIB2; TP53; OBSL1; ATP5S; PTGFR; TMEM63A; DHX9; MAOA; ZBTB1; STC1; BRCC3; ZBTB3; TTBK2; MBTD1; SYNGR2; APH1B; SPAST; TSPAN9; DHX35; DYM; ASL; HIVEP2; STXBP6; ARSD; ATP6V1C1; ZBED4; GPM6B; SPTBN1; PCK2; EGR1; WWTR1; EGR3; APOBEC3F; YES1; APOBEC3G; GADD45B; SLC35E2; PILRB; PARVA; HSPBAP1; NET1; POLA1; MSRA; TPX2; LOC81691; WNK1; ID2; FUBP1; RANBP10; ANP32E; HIST1H4H; ATM; APOBEC3B; DHRS12; TOP2A; HBS1L; FEN1; ZCCHC11; MLF1IP; FASTKD2; CTSZ; RSF1; IDE; KIF11; WASL; MLL; PCSK5; EDA2R; LIMD2; PPP3CB; ZMYM2; SPTLC2; C14ORF1; RSRC1; ENC1; ZMYM6; KIF13A; IDS; DFNB31; AASS; GABPA; RALGPS1; HTATIP2; SREBF1; NCOA2; PARP3; EDN1; BTN3A1; PRMT2; NCOA3; MST1; GAB1; FLJ10038; CACYBP; KIF23; GFRA1; MRPS6; ZFX; C9ORF9; GAL; CTTN; NOV; MDFIC; C9ORF5; KIT; CENPC1; ARHGEF3; ALDOC; ARHGEF7; MATN3; TLR3; DNASE1L1; FAM59A; PTGER4; STX16; HSD17B6; NAPG; ARNTL2; ARNTL; KIAA0754; EXOSC5; RELN; GPC1; HSF2; HSF1; PGGT1B; PCNX; CTNNA1; APBB2; CD58; GPC6; C22ORF29; CBR3; CA12; GINS1; NQO1; SVIL; GINS2; MON2; GCH1; PLEKHA1; YIPF2; NFYA; FAM46C; PEX10; RAB11B; BOP1; RSL1D1; SPCS3; KITLG; KLHL7; MARS; DLC1; TRIP13; F2RL2; MAD2L1; ZWILCH; ZFAND6; HFE; BUB1B; PRDM2; NUCKS1; UBE3B; MANEA; DOCK10; RGS5; FLJ13197; NUSAP1; ENPP1; BAALC; SIRPA; TMEM38B; CD97; TMOD1; KIAA0090; MSX2; LIG1; FBXW11; LIG4; LMO7; PDGFRL; ALDH3A2; ADAM15; GPD2; PPIG; HIST1H2BG; PPIA; STAM2; FBN2; DDX6; IDI1; COPA; RBM8A; YTHDC2; SEL1L; SLC1A1; PSEN2; ARHGAP19; SLC1A4; CACNA1C; LPP; FUT4; NUP160; EFNB2; ARHGAP22; CCNB2; EBP; CCNB1; HRH1; COX11; TASP1; KIF3C; MSC; LONRF1; WNT2; MYH10; SMAD1; MGEA5; SMAD4; CPSF6; HSPA4; RARRES1; GGH; KLHL24; SMAD5; SUZ12P; ANKHD1; ISG20; AHI1; NR4A1; IL6; BMP2; CPS1; DLG5; TRPV4; PHF14; LSM14B; BRWD1; ZCCHC4; ZCCHC2; LRP12; RERE; SNED1; PHTF2; DDX3X; DYRK2; ATP2A2; SLC2A3; RND2; SLC2A6; GPHN; SEC61A2; SENP6; TMEM100; FLCN; SENP7; SNAPC2; SNAPC3; DBT; LRRTM2; AOX1; CASP2; PDE4A; MAN1A1; LRRFIP1; MYBL1; ABCD1; RBM5; SCYL2; SNTA1; ARHGEF11; TGIF2; PTGIS; DDX58; DCAKD; PLA2G4C; SHROOM2; PPP2R5C; QRSL1; LSS; ETV5; TGFBR1; TGFBR2; RBL2; SETBP1; EEF1D; NUP50; RASA2; MVD; INA; CUEDC1; RBKS; DCTN5; MVK; RNF6; HMGCR; TFPI; NR2C1; AURKA; RMND5A; PALMD; TDG; TAF1A; FAM63B; PDLIM5; CLASP2; REPS1; ATF7IP; SPEN; GALNT6; TTC12; RRM2; C4ORF29; USP20; EYA2; PCDHGC3; ATP2B4; ZNF180; PDE4DIP; MAPK14; CLCN7; GALC; GALE; WEE1; SCD; LPIN1; MBTPS2; OTUD3 |
